# Fall-Related Injury in Older Adult Home Care Recipients: A Descriptive Population Study

**DOI:** 10.1093/geroni/igab046.2900

**Published:** 2021-12-17

**Authors:** Danilla Xing, Aleksandra Zecevic, Nicolette Lappan, Yu Ming

**Affiliations:** 1 Western University, London, Ontario, Canada; 2 University of Western Ontario, University of Western Ontario, Ontario, Canada

## Abstract

Canada is experiencing a growing aging population leading to an increase in the number of individuals receiving home care. More needs to be known about home care clients who experience fall-related injuries. The purpose of this study was to describe the characteristics of Ontario home care recipients (65 and older) who experienced fall-related injury, and the characteristics of those injuries. We conducted a population-based descriptive study using secondary data from the IC/ES data repository for the period of 2010-2014. Person-level characteristics were extracted from the Resident Assessment Instrument - Home Care and injury characteristics from ICD-10 CA codes for falls (W00-W19) in combination with injuries (S00-S99 or T00-T14), available from the NACRS database. Descriptive statistics and rates were calculated using R. Results show the population (N= 88,731) was primarily female (67.0%), the largest age group was 85-89 years old (25.5%) and hypertension was the most prevalent (83.0%) chronic condition. Clinical Assessment Protocols (CAPs) indicated need for support in management of IADLs (75.4%), falls (72.3%) and pain (70.3%). Most patients (55.8%) used nine or more medications. In 90 days prior to home care assessment, 39.6% experienced no falls, 32.4% fell once, and 26.1% fell two or more times. Injuries primarily took place within the home (38.2%). Factures were the predominant injury type (40.8%), followed by superficial injuries (19.7%). These findings create a foundation for fall-related injury prevention in home care and further research on risk identification, the efficacy of CAPs, and home environment adjustments.

